# Earnings, job satisfaction, and turnover of nurse practitioners across employment settings

**DOI:** 10.1093/haschl/qxad044

**Published:** 2023-09-14

**Authors:** Joseph G P Hnath, Betty Rambur, David C Grabowski

**Affiliations:** Department of Health Care Policy, Harvard University, Boston, MA 02115, United States; College of Nursing, University of Rhode Island, Kingston, RI 02881, United States; Department of Health Care Policy, Harvard University, Boston, MA 02115, United States

**Keywords:** nurse practitioners, advanced practice registered nurses, nursing workforce, health care workforce, health care labor markets, long-term care

## Abstract

Nurse practitioners (NPs) are an important part of the health care workforce. However, little information is available on NP earnings, job satisfaction, or turnover. National survey data from 2018 offer a pre–COVID-19 baseline for ongoing NP workforce monitoring. We found evidence that NPs earned approximately $92 500 annually, ranging from $82 800 in long-term care to $95 600 in hospital settings. Wages increased with tenure in the workforce and varied considerably by geography. Approximately 1 in 5 NPs switched jobs annually, with some net in-flow to ambulatory settings. Both NPs who left their position or considered leaving reported better pay and benefits, burnout, management role, stressful work environment, career advancement, and inadequate staffing as the primary explanations. These findings were augmented by analysis of 2012–2022 Bureau of Labor Statistics data that illustrated substantial growth in the NP workforce. Improving NP job satisfaction has the direct benefit of supporting a critical and growing segment of the health workforce; it has the additional benefit of reducing job turnover and the associated costs, potentially increasing earnings for NPs. Policies that improve working conditions for NPs in different employment settings will not just increase immediate job satisfaction but also ideally strengthen the longer-term labor market to improve patient outcomes.

## Introduction

The United States currently has a major shortage of primary care physicians.^1-4^ Nurse practitioners (NPs) serve as a potential solution to address this gap, especially in rural areas.^5,6^ Nurse practitioners are the most common group of advanced-practice registered nurses, and studies suggest that they provide primary care that is of comparable quality to that of physicians.^7-9^ Over the period 2010–2017, the number of NPs in the United States more than doubled, from approximately 91 000 to 190 000.^5^ The Bureau of Labor Statistics estimates 2021 national NP employment to be 247 000 and projects employment to grow by 45% by 2031, the fastest growing of all occupations measured.^10^

Nurse practitioners work in various employment settings, including hospitals, ambulatory settings, and long-term care. We currently have a limited understanding of why NPs are entering and exiting these different settings. Two possible explanations are pay and job satisfaction. A National Academies 2021 report found differences in earnings for NPs across employment settings, but the analysis did not account for other potential differences in the characteristics of NPs that could affect wages, such as training and experience.^11^

Similarly, job satisfaction and burnout by NPs, which have been magnified by the hardships from the COVID-19 pandemic, may also differ across employment settings. Specifically, many NPs are considering leaving direct-care roles.^12,13^ A recent survey found that the number of job openings for NPs passed that of MDs for the first time in 28 years.^14^ Previous research on the health care workforce suggests a strong relationship between job dissatisfaction and turnover, and that promotion and training opportunities may play a larger role than the often-focused-on aspects of workload or earnings.^15^ Prior studies have not considered how job satisfaction and burnout for NPs differ across employment settings.

In this study, we examined the factors associated with the supply of NPs in the hospital, ambulatory, and long-term-care settings. Using nationally representative survey data, we consider how worker characteristics, earnings, and job satisfaction differ across these settings. We also examined job transitions across sectors by NPs and the reasons that NPs left or considered leaving their positions.

## Data and methods

Our primary data source was the 2018 National Sample Survey of Registered Nurses (NSSRN), which surveyed more than 100 000 registered nurses (RNs) from approximately 4.6 million licensure records provided by State Boards of Nursing.^16^ Since the 1970s, the NSSRN has been considered the cornerstone of nursing workforce data, utilizing the expertise of the Census Bureau for accurate and secure administration.^17^ Approximately 25 796 NPs were included in this sample, which was representative of 312 673 NPs nationally after accounting for survey weights. Using complex survey methodology to address nonresponse bias, we generated estimates that were nationally representative. All of our variance estimates used the jackknife replication method, and additional information for the survey design can be found in the NSSRN technical documentation.^18^ The scope, depth, accuracy, and national representativeness of the survey due to its longevity, funding support from the Health Resources & Services Administration (HRSA), and expert administration by the Census Bureau are major strengths compared with other surveys on NPs.

Our main outcome measure was annual earnings from the NPs' primary employment. This measure was self-reported in the survey using a free-response entry method, which provided more granular information compared with other data sources with binned measures of earnings. Specifically, respondents were told to report earnings from their “primary nursing position” and to include overtime and bonuses but not sign-on bonuses. Primary nursing position was defined as the nursing position in which they spent the largest share of their working hours. To assess robustness to other statistical assumptions, we compared this measure with log-transformed annual earnings, hourly wage, and log-transformed hourly wage.

Our main independent variable was a mutually exclusive, 4-level category measure of the employment setting that the NP was practicing in, which we categorized as “Hospital”, “Clinic/Ambulatory”, “Long-term Care”, and “Other Setting”. The NSSRN asked respondents to describe the employment setting of their primary employment, and we included the following categories under long-term care: “Nursing home unit not in hospital”, “Rehabilitation facility/long-term care”, “Home health agency/service”, and “Hospital nursing home unit”. We justify our inclusion of home health in long-term care based on definitions of long-term care from the National Institute on Aging^19^ and from the World Health Organization.^20^ Hospital nursing home units may be quite different from the other employment settings, but given that they make up a small share of our sample, our results were similar when we excluded them. Additional information on employment setting and alternative classification measures is shown in Appendix Table SA1.

Our primary analyses consisted of regression analyses examining the relationship between 2017 annual primary earnings for NPs and employment setting (hospital, clinic/ambulatory, long-term care, other). Because characteristics that affected earnings may also be correlated with the employment setting in which the NP worked, we included covariates such as hours worked, its quadratic term, and census region. A major advantage of these data compared with other sources is that we were also able to include a comprehensive list of individual-level characteristics, such as age, gender, race, ethnicity, marital status, children, years of working experience as an NP, education, licensing, previous work experience in health care, self-reported time spent working on patient care, and tenure in current job. We have included the full specification and related information in Appendix S.

Additionally, we described employment changes by including information from the surveys about NPs' 2016 employment status. Specifically, we constructed measures of the proportion of NPs in each of our 4 health care employment settings who experienced a job change. Specifically, we calculated how many NPs changed jobs but remained in the same employment setting, switched to another setting, or were no longer working.

Last, we aggregated the primary reasons that NPs listed for changing employment or for considering an employment change. The full list consisted of about 20 categories of reasons including pay and benefits, burnout, inadequate staffing, and stressful work environment. We documented the reasons listed most often, how often they were listed, and if the reasons were different between those who did change jobs and those who considered an employment change.

We supplement these main analyses with national trends in NP employment from the Bureau of Labor Statistics Occupational Employment and Wage Statistics (BLS OEWS) from 2012–2022. The BLS OEWS is a semiannual survey in collaboration with state workforce agencies of a panel of over 1 million establishments to obtain national estimates.^21^

The data were analyzed using R (version 4.2.3; R Foundation for Statistical Computing), making significant use of the “survey”, “srvyr”, “modelsummary”, “gt”, “marginaleffects”, and “tidyverse” collection of packages.^22-27^

### Limitations

Our study had several limitations. First, our data were from the 2018 NSSRN. The health care system has experienced substantial change since this period due to the COVID-19 pandemic. However, we assert that the broader findings from our study are still generalizable to the present day, and we hope that this encourages newer and ongoing data collection so that we can continually monitor the state of the NP workforce. Second, the NSSRN has a response rate of 50%, so there could be some additional bias; however, this is much better than comparable surveys, such as the 2020 American Association of Nurse Practitioners (AANP) National NP Sample Survey (NNPSS), with a 7% response rate.^16,28^ Third, we were unable to make claims about the causal effects of employment setting on earnings due to unobserved factors that might be associated with choice of setting and wages. Despite this, we controlled for many characteristics that were associated with both employment setting and earnings, and our descriptive findings are important in documenting differences in earnings and NP characteristics across settings.

## Results

**Figure 1.**
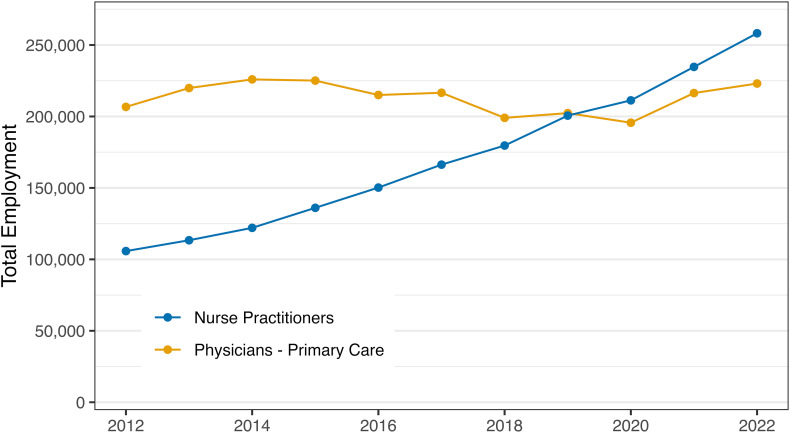
Total employment has increased more for NPs compared with primary care physicians. Source: Authors’ analysis of 2012–2022 BLS OEWS data. Primary Care Physician Specialties: Family, General, Internal, Pediatrics, and OB/GYN. Abbreviations: BLS OEWS, Bureau of Labor Statistics Occupational Employment and Wage Statistics; NP, nurse practitioner.

Although NPs do not exclusively provide primary care, they have become an important part of this workforce. In 2020, 88.9% of NPs were certified in an area of primary care.^28^ Using data from the Bureau of Labor Statistics, the total employment of primary care physicians remained stable from 2012–2022, while total employment for NPs has consistently and significantly increased (Figure 1).

**Figure 2.**
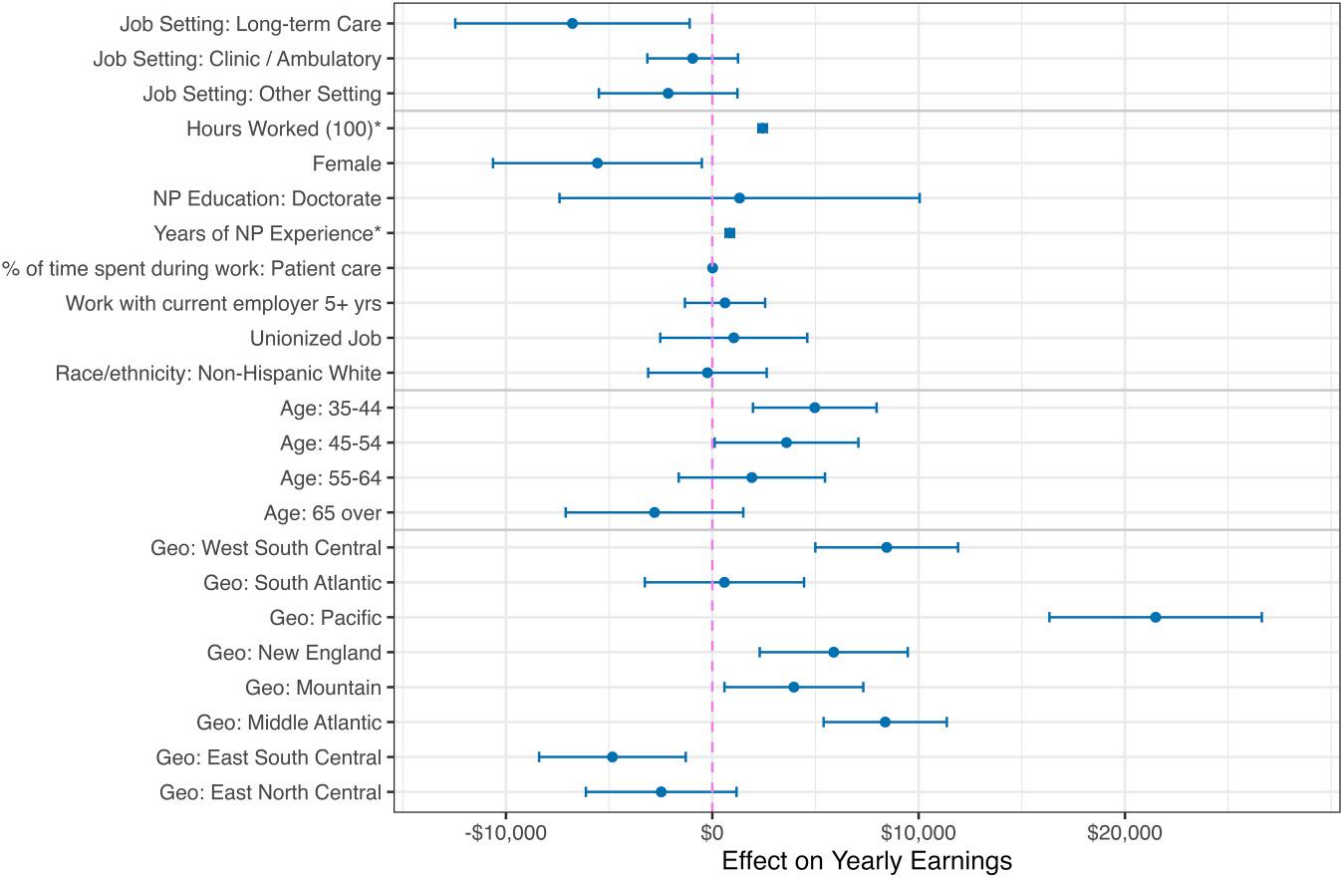
Characteristics affecting NP earnings. Source: Authors’ analysis of 2018 NSSRN data. These are selected coefficients from the main regression model, including 95% CIs. The full table of coefficients can be found in Appendix Table SA2. The reference level is a male, hospital-based NP, age under 35, in the West North Central Census Division (ie, IA, MO, KS, NE, SD, ND, MN). *Due to the nonlinear quadratic terms, these are estimated average contrasts to facilitate comparisons with the linear estimates. Abbreviations: Geo, geographic region; NP, nurse practitioner; NSSRN, National Sample Survey of Registered Nurses.

Given the growing importance of NPs within the primary care workforce, we next examined the association between earnings and working in different settings (Figure 2, Appendix Table SA3). We found a difference in earnings of nearly $10 000 between hospital-based NPs and those in long-term care, with smaller and not statistically significant differences for NPs in ambulatory care or other settings. When controlling for individual-level characteristics that have been documented to affect earnings in similar and other contexts,^5,29,30^ this wage differential decreased to approximately $6000. Additional information on the variation in these characteristics across employment settings is provided in Appendix Table SA2. We found that female NPs made approximately $5600 less, despite already controlling for home-life differences (eg, marriage and children), work experience, and hours worked. Previous studies have found a gender wage gap over twice this size in a slightly different context,^31^ but controlling for hours worked has been found to be an important predictor in other more recent analyses of the gender wage gap.^32^

The number of years since NP graduation (our measure of work experience) was a very strong predictor, where an NP made approximately $8000 more after 5 years of experience compared with their first job (zero years of experience), but only approximately $9000 more from that when comparing 5 years with 20 years of work experience. We did not find evidence that working at the same job for 5 or more years, having a union job, or being a racial or ethnic minority had a significant effect on earnings.

Some of the largest differentials were across geographic areas. Our reference geography level was the West North Central Census Division (ie, IA, MO, KS, NE, SD, ND, MN). By comparison, NPs working in the Pacific region made approximately $21 000 more, NPs in the mid-Atlantic and West South-Central regions made approximately $8000 more, NPs in the New England region made approximately $6000 more, and NPs in the East South-Central region made approximately $5000 less. These area wage differences were likely influenced by regional labor market and regulatory factors. However, when we included within-region indicators of 3 levels of NP practice authority regulation,^33^ we did not find evidence that these practice authority regulations influence wages.

Our main results were robust to other changes in our regression model specifications, such as transforming the independent variable to logged values, using hourly earnings instead of total annual earnings, transforming hourly earnings to logged values, and removing hours worked as a dependent variable. These results can be found in Appendix Table SA4. We classified hospital-based ambulatory care as hospital-based rather than ambulatory care, but Appendix Table SA5 shows that our results are not sensitive to this decision.

Nurse practitioner earnings in long-term-care jobs were not uniform. In particular, the work environment for home health was quite different from other facility-based jobs. Specifically, the overall wage differential that we documented in Figure 2 was mostly driven by the lower earnings of home health NPs.

**Figure 3.**
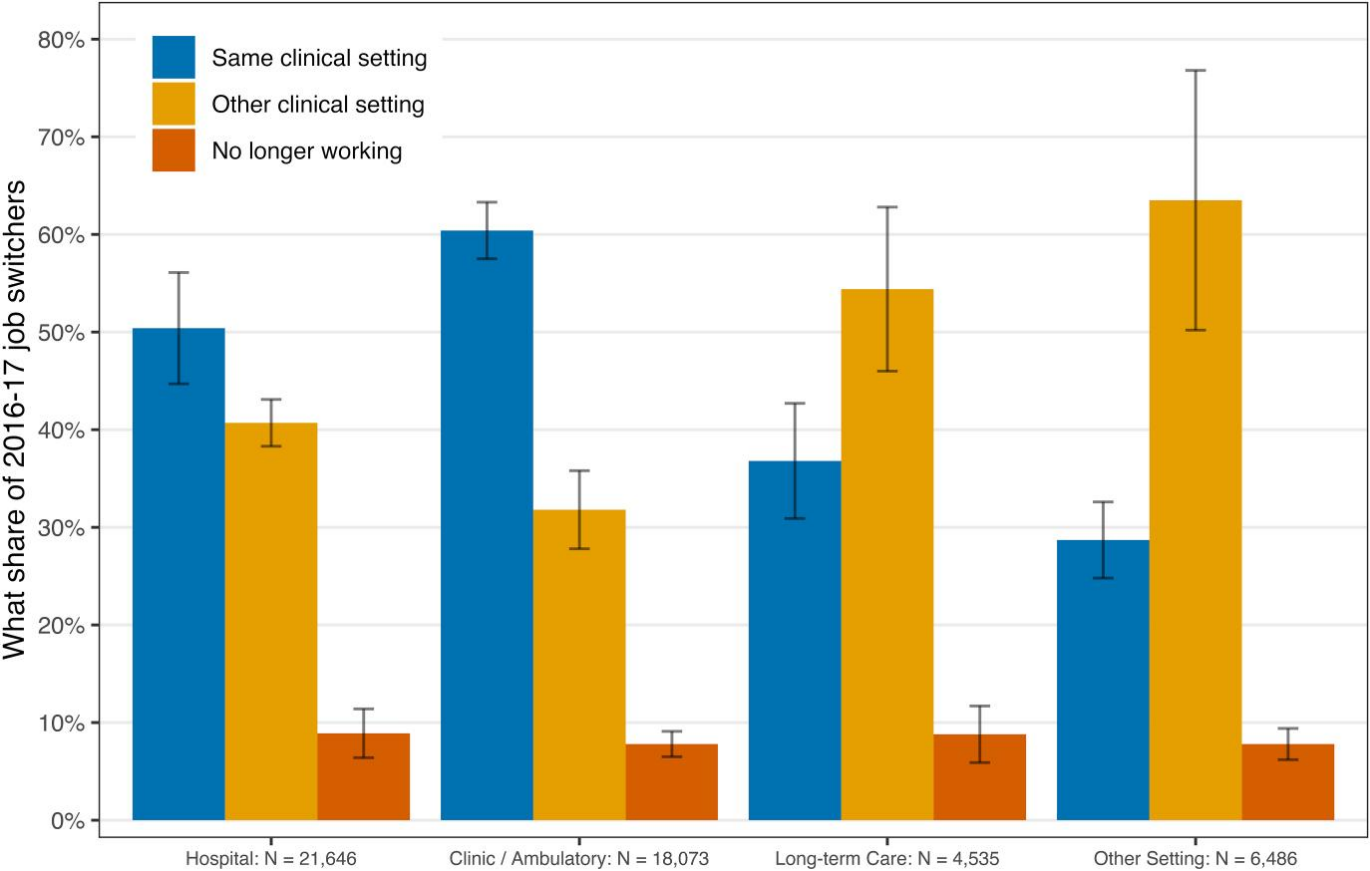
Nurse practitioners changing jobs sometimes also change setting. Source: Authors’ analysis of 2018 NSSRN data. This sample is for the subset of NPs who changed jobs from 2016 to 2017 (∼20%). Uncertainty from the complex survey design is shown with 1-SE bars. Abbreviations: NP, nurse practitioner; NSSRN, National Sample Survey of Registered Nurses.

Nurse practitioners changed jobs relatively frequently (see Figure 3). Although over half of NPs in hospital and ambulatory settings who changed jobs remained in that general setting, that was only the case for approximately one-third of those working in long-term care or other settings. Among job switchers, which represented approximately 20% of the total NP population in our sample, we observed net in-flows to the ambulatory setting and net out-flows from the other 3 settings, but we observed NPs moving across all 4 employment setting categories. Additionally, we found no evidence of differential exit from the workforce across settings.

**Figure 4.**
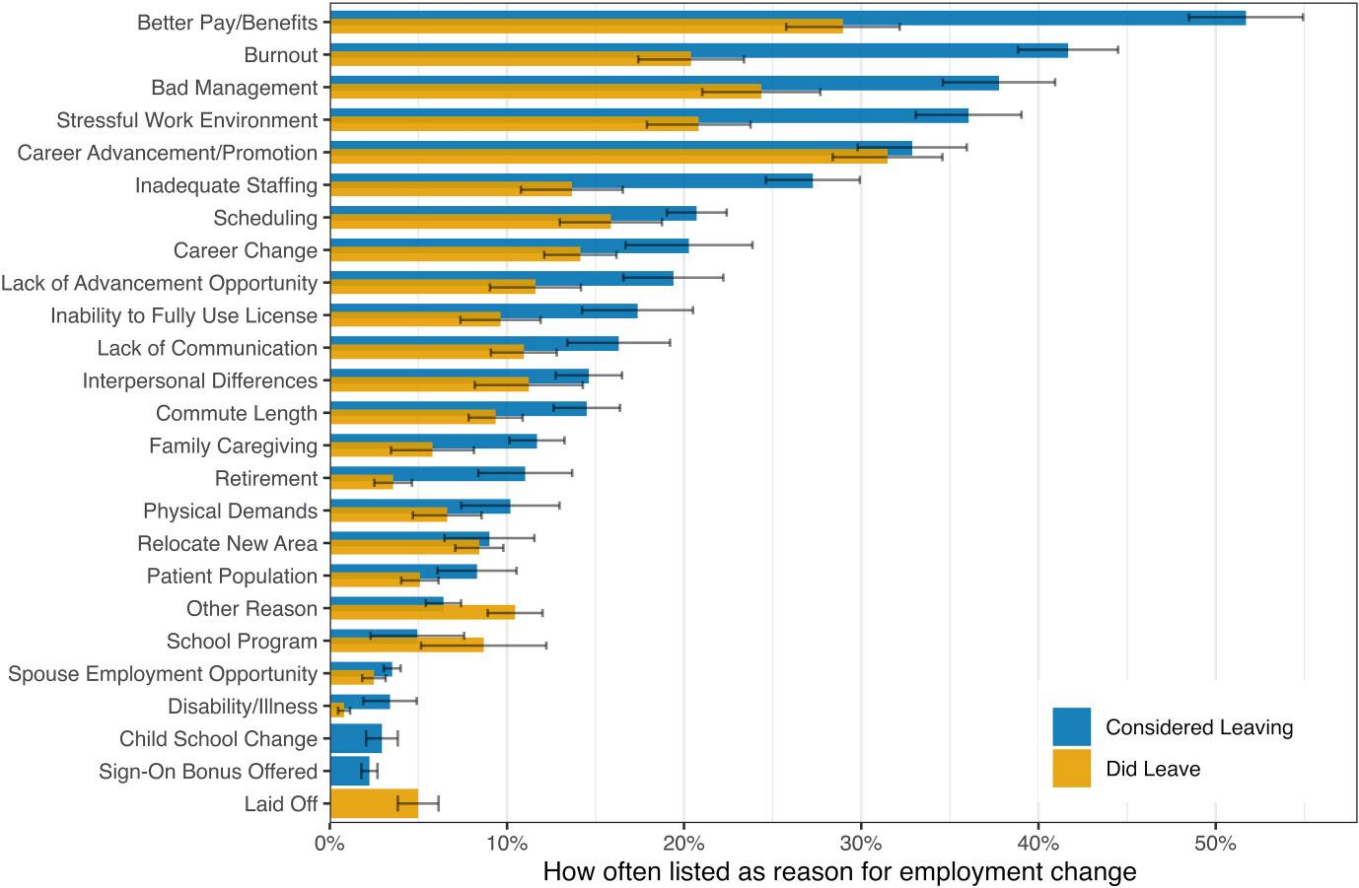
What influences employment change for nurse practitioners? Source: Authors’ analysis of 2018 NSSRN data. Results for NPs who did leave (2016) and for those who considered leaving (2017) do not necessarily include all surveyed NPs if either question was not applicable. The denominator is NPs who considered or did leave their job, not all NPs. We display 95% CI error bars due to the complex survey design. Abbreviations: NP, nurse practitioner; NSSRN, National Sample Survey of Registered Nurses.

The NPs listed similar reasons for considering employment change compared with reasons listed by NPs who did change employment (see Figure 4). Although over half of all NPs who considered leaving listed better pay or benefits as a reason, less than 30% of NPs who did change employment listed this reason. Career advancement was listed a little over 30% of the time for both groups. Burnout, stressful work environment, and inadequate staffing were listed for more than one-quarter of NPs who considered changing jobs but these reasons were all reduced to approximately half of those levels for NPs who did change jobs. There was some suggestive evidence of differences across clinical locations, such as hospital NPs focusing on career advancement about twice as often whereas long-term-care NPs specified stressful work environments 50% more often.

## Discussion

As the population ages, demand for health care is increasing, requiring a greater supply of health care workers, including NPs.^11^ This study considered how wages vary for NPs across settings and how pay and other factors related to NP burnout and exit from the workforce. We found that NPs in hospital settings were paid better than their counterparts in other settings, even after controlling for a series of individual characteristics. Among those NPs who left their jobs, there was a net out-flow away from the lower-paying sectors and towards the higher-paying hospital sector. In our analysis of factors associated with why NPs left their job or considered leaving their position, better pay was the most cited factor. Other important factors included burnout and leaving to seek a management role. Although the relationship between burnout and quality of care is complex, better staffing and working environments were associated with nursing resilience in response to the COVID-19 pandemic.^34-36^

Previous work has shown broad heterogeneity in earnings for health care workers across different employment settings, such as in a hospital or clinic.^11,37,38^ Specifically for NPs, previous work has documented high-level variation in earnings across employment settings, and specific differences for primary care NPs.^5,11,28,39^ However, most existing findings do not specifically look at long-term-care NPs, and the National Academies report found much smaller differences when using the same data,^11^ suggesting the importance of accounting for individual-level characteristics. Moreover, other studies have documented high turnover among long-term-care workers.^40-42^ However, we believe our study is the first to examine these issues jointly with individual-level data. If long-term-care settings and other health care providers are going to compete with hospitals for NPs, they need to identify ways to improve pay and other job attributes valued by workers.

A key issue underlying discrepancy in NP pay is the mix of payers across the various settings. Hospitals rely predominantly on commercial insurance and Medicare, while long-term-care providers rely predominantly on Medicaid and Medicare.^43^ Even though most long-term-care recipients have their health care covered by Medicare, long-term-care providers often have fewer resources to hire staff directly due to the large role of Medicaid. Medicare Advantage institutional special needs plans (or I-SNPs) are one innovative Medicare payment model to recruit NPs directly to care for long-stay nursing home residents covered mainly by Medicaid.^44^ However, I-SNPs are still only present in a relatively small share of US nursing homes.

Although better pay may be necessary for recruiting new workers, it is certainly not sufficient. Similar to work focusing on RNs,^45,46^ our study suggests other job factors matter to NPs. A recent survey found that 80% of nurses experienced racism or discrimination from patients, while 60% experienced this from colleagues, leading to worse mental health and well-being.^47^ Our findings indicate that approximately half of NPs who considered leaving their current employment cited pay and benefits as a reason. Although this is the largest category, half of NPs who considered leaving their job did not list their earnings as a reason why. Compared with other industries, health care workers may have chosen this profession primarily to care for others, in addition to making a living. Leveraging this intrinsic motivation of health care workers to care for patients should be a priority for policymakers.^48^ A recent paper focused on improving the nursing workforce in response to COVID-19 proposed a framework that emphasizes many different improvements in the workplace environment.^49^

Our study is the first to examine individual-level changes in NP employment, suggesting that NPs in ambulatory care settings who changed jobs were most likely to stay in the ambulatory care setting, whereas those in long-term care and other settings are much less likely to stay in their setting. Although these “across-setting” job changes were not extremely common, they indicated that the preferences NPs may have for working in different clinical environments are not fixed. Caring for patients in a hospital is quite different from an ambulatory setting, which are both quite different from that in a long-term-care setting. This is due to the types of patients, the most prevalent health conditions, pace of care, and environment of other health care workers. As long-term care becomes an increasingly important part of the overall health care system, it would be prudent to carefully evaluate the reasons why NPs leave long-term-care settings and what can be done to improve their satisfaction with these jobs. Concerns about staffing and substandard conditions for patients have been identified by other studies as key problems in this sector.^50,51^ This effort is particularly salient, given findings that deployment of NPs in long-term care was associated with a decrease in unnecessary hospitalizations, increased access to health care, and improved health outcomes.^52^

## Conclusion

Taken as a whole, our study illustrates marked discrepancies in NP earnings by gender, turnover by setting, and job satisfaction. Given that approximately half of NPs leave their position for career advancement and better compensation, organizations aiming to create such opportunities have the potential to improve patient outcomes and reduce strain and burnout in the health care labor market. Ongoing analysis of this growing share of the US health care workforce is warranted.

## Supplementary Material

qxad044_Supplementary_Data

## References

[1] Zhang X, Lin D, Pforsich H, Lin VW. Physician workforce in the United States of America: forecasting nationwide shortages. Hum Resour Health. 2020;18(1):8. 32029001 10.1186/s12960-020-0448-3 PMC7006215

[2] Kirch DG, Petelle K. Addressing the physician shortage: the peril of ignoring demography. JAMA. 2017;317(19):1947. 28319233 10.1001/jama.2017.2714

[3] Scheffler RM, Arnold DR. Projecting shortages and surpluses of doctors and nurses in the OECD: what looms ahead. Health Econ Policy Law. 2019;14(2):274–290. 29357954 10.1017/S174413311700055X

[4] Bodenheimer TS, Smith MD. Primary care: proposed solutions to the physician shortage without training more physicians. Health Aff (Millwood). 2013;32(11):1881–1886. 24191075 10.1377/hlthaff.2013.0234

[5] Auerbach DI, Buerhaus PI, Staiger DO. Implications of the rapid growth of the nurse practitioner workforce in the US. Health Aff (Millwood). 2020;39(2):273–279. 32011941 10.1377/hlthaff.2019.00686

[6] Barnes H, Richards MR, McHugh MD, Martsolf G. Rural and nonrural primary care physician practices increasingly rely on nurse practitioners. Health Aff (Millwood). 2018;37(6):908–914. 29863933 10.1377/hlthaff.2017.1158 PMC6080248

[7] Naylor MD, Kurtzman ET. The role of nurse practitioners in reinventing primary care. Health Aff (Millwood). 2010;29(5):893–899. 20439877 10.1377/hlthaff.2010.0440

[8] Laurant M, van der Biezen M, Wijers N, Watananirun K, Kontopantelis E, van Vught AJ. Nurses as substitutes for doctors in primary care. Cochrane Database Syst Rev. 2018;7:CD001271. 10.1002/14651858.CD001271.pub3 PMC6367893 30011347

[9] Barnett M, Balkissoon C, Sandhu J. The level of quality care nurse practitioners provide compared with their physician colleagues in the primary care setting: a systematic review. J Am Assoc Nurse Pract. 2022;34(3):457–464. 34678807 10.1097/JXX.0000000000000660

[10] US Bureau of Labor Statistics. Fastest growing occupations. Accessed August 8, 2023. https://www.bls.gov/emp/tables/fastest-growing-occupations.htm

[11] Committee on the Future of Nursing 2020–2030, National Academy of Medicine, National Academies of Sciences, Engineering, and Medicine. Chapter 3 - The nursing workforce. In: Wakefield MK, Williams DR, Menestrel SL, Flaubert JL, eds. The Future of Nursing 2020-2030: Charting a Path to Achieve Health Equity. National Academies Press; 2021:59–98. 34524769

[12] Faraz Covelli A, Barnes H. Novice nurse practitioners’ employment decisions and role transition experiences during COVID-19. J Prof Nurs. 2023;47:81–87. 37295916 10.1016/j.profnurs.2023.05.009 PMC10202406

[13] Berlin G, Lapointe M, Murphy M. Surveyed nurses consider leaving direct patient care at elevated rates. Published 2022. Accessed August 17, 2023. https://www.mckinsey.com/industries/healthcare/our-insights/surveyed-nurses-consider-leaving-direct-patient-care-at-elevated-rates#/

[14] Merritt Hawkins. 2021 Review of physician and advanced practitioner recruiting incentives. Accessed June 8, 2023. https://www.merritthawkins.com/trends-and-insights/article/reports/2021-review-of-physician-and-advanced-practitioner-recruiting-incentives/

[15] Shields MA, Ward M. Improving nurse retention in the National Health Service in England: the impact of job satisfaction on intentions to quit. J Health Econ. 2001;20(5):677–701. 11558644 10.1016/s0167-6296(01)00092-3

[16] US Department of Health and Human Services, Health Resources and Services Administration, National Center for Health Workforce Analysis. Technical report for the national sample survey of registered nurses, Rockville, Maryland. 2019. Accessed August 17, 2023. https://bhw.hrsa.gov/sites/default/files/bureau-health-workforce/data-research/nssrn-technical-report.pdf

[17] US Census Bureau. Frequently asked questions. Accessed August 8, 2023. https://www.census.gov/programs-surveys/nssrn/about/faq.html

[18] National Sample Survey of Registered Nurses (NSSRN) | Bureau of Health Workforce. Accessed May 12, 2023. https://bhw.hrsa.gov/data-research/access-data-tools/national-sample-survey-registered-nurses

[19] National Institute on Aging. What is long-term care? Accessed August 17, 2023. https://www.nia.nih.gov/health/what-long-term-care

[20] World Health Organization. Long-term care. Accessed August 17, 2023. https://www.who.int/europe/news-room/questions-and-answers/item/long-term-care

[21] US Bureau of Labor Statistics. Occupational employment and wage statistics. Accessed August 1, 2023. https://www.bls.gov/oes/

[22] Lumley T. Complex Surveys: A Guide to Analysis Using R. John Wiley; 2010.

[23] Freedman Ellis G, Schneider B. srvyr: ‘dplyr’-Like syntax for summary statistics of survey data. Published online 2023. Accessed August 17, 2023. https://CRAN.R-project.org/package=srvyr

[24] Arel-Bundock V. Modelsummary: data and model summaries in R. J Stat Softw. 2022;103:1–23.

[25] Iannone R, Cheng J, Schloerke B, Hughes E, Lauer A, Seo J. gt: Easily create presentation-ready display tables. Published online 2023. Accessed August 17, 2023. https://CRAN.R-project.org/package=gt

[26] Arel-Bundock V. marginaleffects: Predictions, comparisons, slopes, marginal means, and hypothesis tests. Published online 2023. Accessed August 17, 2023. https://CRAN.R-project.org/package=marginaleffects

[27] Wickham H, Averick M, Bryan J, et al Welcome to the Tidyverse. J Open Source Softw. 2019;4(43):1686.

[28] *2020 AANP National NP Sample Survey*. American Association of Nurse Practitioners; 2021. Accessed August 17, 2023. https://storage.aanp.org/www/documents/no-index/research/2020-NP-Sample-Survey-Report.pdf

[29] Lemieux T. The “mincer equation” thirty years after schooling, experience, and earnings. In: Grossbard S, ed. Jacob Mincer A Pioneer of Modern Labor Economics. Kluwer Academic Publishers; 2006:127–145.

[30] Wagner LM, Bates T, Spetz J. The association of race, ethnicity, and wages among registered nurses in long-term care. Med Care. 2021;59(Suppl 5):S479–S485. 34524246 10.1097/MLR.0000000000001618 PMC8428870

[31] Greene J, El-Banna MM, Briggs LA, Park J. Gender differences in nurse practitioner salaries. J Am Assoc Nurse Pract. 2017;29(11):667–672. 28857491 10.1002/2327-6924.12512

[32] Denning JT, Jacob BA, Lefgren LJ, vom Lehn C. The return to hours worked within and across occupations: implications for the gender wage gap. ILR Rev. 2022;75(5):1321–1347.

[33] Phillips SJ. 29th Annual APRN legislative update. Nurse Pract. 2017;42(1):18–46. 10.1097/01.NPR.0000511006.68348.93 28002144

[34] Vahey DC, Aiken LH, Sloane DM, Clarke SP, Vargas D. Nurse burnout and patient satisfaction. Med Care. 2004;42(2 Suppl):II57–II66. 14734943 10.1097/01.mlr.0000109126.50398.5a PMC2904602

[35] Casalino LP, Li J, Peterson LE, et al Relationship between physician burnout and the quality and cost of care for Medicare beneficiaries is complex: study examines the relationship between physician burnout and the quality and cost of care for Medicare beneficiaries. Health Aff (Millwood). 2022;41(4):549–556. 35377764 10.1377/hlthaff.2021.00440 PMC9934398

[36] Aiken LH, Sloane DM, McHugh MD, Pogue CA, Lasater KB. A repeated cross-sectional study of nurses immediately before and during the COVID-19 pandemic: implications for action. Nurs Outlook. 2023;71(1):101903. 36588039 10.1016/j.outlook.2022.11.007 PMC9729649

[37] US Bureau of Labor Statistics. Registered nurses: occupational outlook handbook. Accessed December 8, 2021. https://www.bls.gov/ooh/healthcare/registered-nurses.htm#tab-6

[38] US Bureau of Labor Statistics. Licensed practical and licensed vocational nurses. Accessed May 12, 2023. https://www.bls.gov/oes/current/oes292061.htm

[39] Li Y, Holmes GM, Fraher EP, Mark BA, Jones CB. Primary care nurse practitioner wage differences by employment setting. Nurs Outlook. 2018;66(6):528–538. 30104024 10.1016/j.outlook.2018.06.009

[40] Gandhi A, Yu H, Grabowski DC. High nursing staff turnover in nursing homes offers important quality information: study examines high turnover of nursing staff at US nursing homes. Health Aff (Millwood). 2021;40(3):384–391. 33646872 10.1377/hlthaff.2020.00957 PMC7992115

[41] Matthews M, Carsten MK, Ayers DJ, Menachemi N. Determinants of turnover among low wage earners in long term care: the role of manager-employee relationships. Geriatr Nur (Lond). 2018;39(4):407–413. 10.1016/j.gerinurse.2017.12.004 29499899

[42] Hayhurst A, Saylor C, Stuenkel D. Work environmental factors and retention of nurses. J Nurs Care Qual. 2005;20(3):283–288. 15965395 10.1097/00001786-200507000-00015

[43] Grabowski DC. Medicare and Medicaid: conflicting incentives for long-term care. Milbank Q. 2007;85(4):579–610. 18070331 10.1111/j.1468-0009.2007.00502.x PMC2690349

[44] McGarry BE, Grabowski DC. Managed care for long-stay nursing home residents: an evaluation of institutional special needs plans. Am J Manag Care. 2019;25(9):438–443. 31518093

[45] McHugh MD, Wage C. Wage, work environment and staffing: effects on nurse outcomes. Policy Polit Nurs Pract. 2014;15(3-4):72–80. 25121923 10.1177/1527154414546868 PMC4667784

[46] McHugh MD, Kutney-Lee A, Cimiotti JP, Sloane DM, Aiken LH. Nurses’ widespread job dissatisfaction, burnout, and frustration with health benefits signal problems for patient care. Health Aff (Millwood). 2011;30(2):202–210. 21289340 10.1377/hlthaff.2010.0100 PMC3201822

[47] Robert Wood Johnson Foundation. Insights into nurses’ experiences and perceptions of discrimination. Published online May 2023. Accessed August 17, 2023. https://www.statnews.com/wp-content/uploads/2023/05/rwjf473632.pdf

[48] McWilliams JM. Professionalism revealed: rethinking quality improvement in the wake of a pandemic. NEJM Catal Innov Care Deliv. 2020;1(5):CAT.20.0226.

[49] Buerhaus P, Fraher E, Frogner B, Buntin M, O’Reilly-Jacob M, Clarke S. Toward a stronger post-pandemic nursing workforce. N Engl J Med. 2023;389(3):200–202. 37458259 10.1056/NEJMp2303652

[50] Grabowski DC. Putting the nursing and home in nursing homes. Innov Aging. 2022;6(4):igac029. 10.1093/geroni/igac029 PMC9196684 35712322

[51] Aloisio LD, Coughlin M, Squires JE. Individual and organizational factors of nurses’ job satisfaction in long-term care: a systematic review. Int J Nurs Stud. 2021;123:104073. 34536909 10.1016/j.ijnurstu.2021.104073

[52] Mileski M, Pannu U, Payne B, Sterling E, McClay R. The impact of nurse practitioners on hospitalizations and discharges from long-term nursing facilities: a systematic review. Healthcare (Basel). 2020;8(2):114. 32354015 10.3390/healthcare8020114 PMC7348833

