# Supplementary material for: Earnings, job satisfaction, and turnover of nurse practitioners across employment settings
Source: Health Aff Sch. 2023 Sep 14;1(3):qxad044. doi: 10.1093/haschl/qxad044 (PMC10986281; doi:10.1093/haschl/qxad044)
Supplement: qxad044_Supplementary_Data [file qxad044_Supplementary_Data.zip › appendix - NP earnings and satisfaction.docx]

**Appendix:**

**Earnings, Job Satisfaction, and Turnover of Nurse Practitioners Across Employment Settings**

Additional Information on the data

More information about the NSSRN is at their website: <https://bhw.hrsa.gov/data-research/access-data-tools/national-sample-survey-registered-nurses>

Specifically, here are some exact resources:

- Data: <https://data.hrsa.gov/topics/health-workforce/nursing-workforce-survey-data>
- Technical documentation: <https://bhw.hrsa.gov/sites/default/files/bureau-health-workforce/data-research/nssrn-technical-report.pdf>
- Survey instrument: <https://bhw.hrsa.gov/sites/default/files/bureau-health-workforce/data-research/nssrn-questionaire.pdf>

Because this is publicly available data, all code + data for complete replication is available upon request.

**Additional Regression Information**

Our main earnings regression analyses are of the following design:

$$Annual primary earnings \sim Health care setting + hours worked + hours worked \left( squared \right)+ Census region + female + Full time work + NP experience (years) + NP experience [squared] + NP education (e.g., Master’s, Doctoral) + \% Time spent doing patient care + Married + union job + young children + not young children + race: non-Hispanic White + LPV/LVN license + No previous health-related jobs prior to RN + current employer for at least 5 years + age bins +\varepsilon$$

Where we have three “levels” of regression models with an increasing number of covariates as independent variables. The first model (“basic”) has only the main independent variable of interest, employment setting, in red font. The second (“intermediate”) model adds the covariates in blue font (hours worked, its quadratic term, and Census region), and the third model (“full”) adds a comprehensive list of individual-level characteristics (in green font).

| **Appendix Table A1: Employment setting and care level overlap for NPs** | | | | | | |
| --- | --- | --- | --- | --- | --- | --- |
| Grouped Employment Setting | Care level / type of work | Svy-Wtd: N | Std Err | % Share - Total | % Share - Grouped | Raw Counts |
| Clinic / Ambulatory | AMBULATORY CARE (INCLUDING PRIMARY CARE OUTPATIENT SETTINGS, EXCEPT SURGICAL) | 80,637 | 1,446 | 31.7% | 42.3% | 10,863 |
| Hospital | GENERAL OR SPECIALTY INPATIENT | 29,760 | 1,212 | 11.7% | 39.4% | 3,050 |
| Hospital | AMBULATORY CARE (INCLUDING PRIMARY CARE OUTPATIENT SETTINGS, EXCEPT SURGICAL) | 17,916 | 1,016 | 7% | 39.4% | 10,863 |
| Hospital | CRITICAL/INTENSIVE CARE | 17,711 | 1,572 | 7% | 39.4% | 996 |
| Hospital | EMERGENCY | 13,733 | 2,000 | 5.4% | 39.4% | 672 |
| Other Setting | AMBULATORY CARE (INCLUDING PRIMARY CARE OUTPATIENT SETTINGS, EXCEPT SURGICAL) | 8,061 | 1,286 | 3.2% | 11.2% | 10,863 |
| Clinic / Ambulatory | URGENT CARE | 7,310 | 439 | 2.9% | 42.3% | 883 |
| Long-term Care | HOME HEALTH/HOSPICE | 7,268 | 1,545 | 2.9% | 7.2% | 565 |
| Hospital | SURGERY (INCLUDING AMBULATORY, PRE-OPERATIVE, POST-OPERATIVE, POST-ANESTHESIA) | 6,544 | 571 | 2.6% | 39.4% | 671 |
| Long-term Care | LONG-TERM CARE/NURSING HOME | 6,096 | 459 | 2.4% | 7.2% | 615 |
| Other Setting | EDUCATION | 6,021 | 473 | 2.4% | 11.2% | 774 |
| Clinic / Ambulatory | GENERAL OR SPECIALTY INPATIENT | 5,569 | 426 | 2.2% | 42.3% | 3,050 |
| Clinic / Ambulatory | PUBLIC HEALTH/COMMUNITY HEALTH | 4,430 | 455 | 1.7% | 42.3% | 551 |
| Other Setting | GENERAL OR SPECIALTY INPATIENT | 4,383 | 769 | 1.7% | 11.2% | 3,050 |
| Hospital | STEP-DOWN, TRANSITIONAL, PROGRESSIVE, TELEMETRY | 3,944 | 1,014 | 1.6% | 39.4% | 132 |
| Clinic / Ambulatory | SCHOOL NURSE | 2,950 | 441 | 1.2% | 42.3% | 193 |
| Hospital | HEALTH CARE MANAGEMENT/ADMINISTRATION | 2,817 | 973 | 1.1% | 39.4% | 334 |
| Other Setting | PUBLIC HEALTH/COMMUNITY HEALTH | 2,018 | 1,435 | 0.8% | 11.2% | 551 |
| Clinic / Ambulatory | SURGERY (INCLUDING AMBULATORY, PRE-OPERATIVE, POST-OPERATIVE, POST-ANESTHESIA) | 1,929 | 213 | 0.8% | 42.3% | 671 |
| Other Setting | CARE COORDINATION/PATIENT NAVIGATION | 1,832 | 697 | 0.7% | 11.2% | 217 |
| Other Setting | HEALTH CARE MANAGEMENT/ADMINISTRATION | 1,611 | 601 | 0.6% | 11.2% | 334 |
| Other Setting | OTHER, SPECIFY | 1,597 | 369 | 0.6% | 11.2% | 182 |
| Hospital | EDUCATION | 1,558 | 406 | 0.6% | 39.4% | 774 |
| Clinic / Ambulatory | EDUCATION | 1,279 | 478 | 0.5% | 42.3% | 774 |
| Hospital | ANCILLARY CARE (RADIOLOGY, LABORATORY) | 1,146 | 267 | 0.5% | 39.4% | 169 |
| Hospital | CARE COORDINATION/PATIENT NAVIGATION | 1,121 | 233 | 0.4% | 39.4% | 217 |
| Long-term Care | REHABILITATION | 1,040 | 188 | 0.4% | 7.2% | 129 |
| Other Setting | HOME HEALTH/HOSPICE | 939 | 137 | 0.4% | 11.2% | 565 |
| Hospital | URGENT CARE | 935 | 145 | 0.4% | 39.4% | 883 |
| Long-term Care | AMBULATORY CARE (INCLUDING PRIMARY CARE OUTPATIENT SETTINGS, EXCEPT SURGICAL) | 828 | 156 | 0.3% | 7.2% | 10,863 |
| Clinic / Ambulatory | HEALTH CARE MANAGEMENT/ADMINISTRATION | 780 | 141 | 0.3% | 42.3% | 334 |
| Long-term Care | GENERAL OR SPECIALTY INPATIENT | 778 | 283 | 0.3% | 7.2% | 3,050 |
| Other Setting | RESEARCH | 728 | 132 | 0.3% | 11.2% | 142 |
| Long-term Care | SUB-ACUTE CARE | 677 | 112 | 0.3% | 7.2% | 108 |
| Clinic / Ambulatory | CARE COORDINATION/PATIENT NAVIGATION | 661 | 133 | 0.3% | 42.3% | 217 |
| Hospital | LONG-TERM CARE/NURSING HOME | 565 | 316 | 0.2% | 39.4% | 615 |
| Hospital | REHABILITATION | 552 | 297 | 0.2% | 39.4% | 129 |
| Long-term Care | HEALTH CARE MANAGEMENT/ADMINISTRATION | 483 | 126 | 0.2% | 7.2% | 334 |
| Clinic / Ambulatory | ANCILLARY CARE (RADIOLOGY, LABORATORY) | 457 | 80 | 0.2% | 42.3% | 169 |
| Hospital | RESEARCH | 437 | 84 | 0.2% | 39.4% | 142 |
| Hospital | SUB-ACUTE CARE | 390 | 180 | 0.2% | 39.4% | 108 |
| Long-term Care | EDUCATION | 371 | 156 | 0.1% | 7.2% | 774 |
| Clinic / Ambulatory | LONG-TERM CARE/NURSING HOME | 344 | 97 | 0.1% | 42.3% | 615 |
| Long-term Care | CARE COORDINATION/PATIENT NAVIGATION | 330 | 118 | 0.1% | 7.2% | 217 |
| Hospital | INFORMATICS | 320 | 162 | 0.1% | 39.4% | 20 |
| Clinic / Ambulatory | HOME HEALTH/HOSPICE | 305 | 71 | 0.1% | 42.3% | 565 |
| Hospital | OTHER, SPECIFY | 281 | 140 | 0.1% | 39.4% | 182 |
| Other Setting | LONG-TERM CARE/NURSING HOME | 281 | 81 | 0.1% | 11.2% | 615 |
| Clinic / Ambulatory | OTHER, SPECIFY | 267 | 76 | 0.1% | 42.3% | 182 |
| Other Setting | REHABILITATION | 198 | 89 | 0.1% | 11.2% | 129 |
| Hospital | PUBLIC HEALTH/COMMUNITY HEALTH | 177 | 75 | 0.1% | 39.4% | 551 |
| Hospital | HOME HEALTH/HOSPICE | 158 | 52 | 0.1% | 39.4% | 565 |
| Long-term Care | STEP-DOWN, TRANSITIONAL, PROGRESSIVE, TELEMETRY | 150 | 143 | 0.1% | 7.2% | 132 |
| Other Setting | SCHOOL NURSE | 144 | 56 | 0.1% | 11.2% | 193 |
| Clinic / Ambulatory | SUB-ACUTE CARE | 138 | 48 | 0.1% | 42.3% | 108 |
| Clinic / Ambulatory | EMERGENCY | 135 | 55 | 0.1% | 42.3% | 672 |
| Clinic / Ambulatory | RESEARCH | 132 | 58 | 0.1% | 42.3% | 142 |
| Other Setting | INFORMATICS | 121 | 58 | 0% | 11.2% | 20 |
| Other Setting | CRITICAL/INTENSIVE CARE | 108 | 64 | 0% | 11.2% | 996 |
| Long-term Care | ANCILLARY CARE (RADIOLOGY, LABORATORY) | 107 | 41 | 0% | 7.2% | 169 |
| Other Setting | ANCILLARY CARE (RADIOLOGY, LABORATORY) | 92 | 29 | 0% | 11.2% | 169 |
| Other Setting | SURGERY (INCLUDING AMBULATORY, PRE-OPERATIVE, POST-OPERATIVE, POST-ANESTHESIA) | 90 | 30 | 0% | 11.2% | 671 |
| Long-term Care | OTHER, SPECIFY | 83 | 43 | 0% | 7.2% | 182 |
| Other Setting | EMERGENCY | 77 | 39 | 0% | 11.2% | 672 |
| Other Setting | URGENT CARE | 69 | 26 | 0% | 11.2% | 883 |
| Clinic / Ambulatory | CRITICAL/INTENSIVE CARE | 66 | 26 | 0% | 42.3% | 996 |
| Clinic / Ambulatory | REHABILITATION | 54 | 21 | 0% | 42.3% | 129 |
| Long-term Care | CRITICAL/INTENSIVE CARE | 42 | 25 | 0% | 7.2% | 996 |
| Hospital | SCHOOL NURSE | 39 | 27 | 0% | 39.4% | 193 |
| Other Setting | SUB-ACUTE CARE | 39 | 22 | 0% | 11.2% | 108 |
| Long-term Care | INFORMATICS | 35 | 25 | 0% | 7.2% | 20 |
| Long-term Care | PUBLIC HEALTH/COMMUNITY HEALTH | 34 | 21 | 0% | 7.2% | 551 |
| Clinic / Ambulatory | INFORMATICS | 27 | 21 | 0% | 42.3% | 20 |
| Long-term Care | EMERGENCY | 18 | 19 | 0% | 7.2% | 672 |
| Long-term Care | URGENT CARE | 12 | 10 | 0% | 7.2% | 883 |
| Other Setting | STEP-DOWN, TRANSITIONAL, PROGRESSIVE, TELEMETRY | 8 | 8 | 0% | 11.2% | 132 |
| Long-term Care | RESEARCH | 7 | 7 | 0% | 7.2% | 142 |

| **Appendix Table A2: Nurse Practitioner (NP) Characteristics Vary by Employment Setting** | | | | | |
| --- | --- | --- | --- | --- | --- |
| *Characteristic*  *(% unless otherwise specified)* | *Overall* | *Hospital* | *Clinic / Ambulatory* | *Long-term Care* | *Other Setting* |
| Survey-Weighted Sample Size (n) | 254,349 (4,116) | 100,104 (3,519) | 107,468 (1,848) | 18,360 (1,606) | 28,417 (2,401) |
| Total earnings from primary job ($) | 92,495 (1,107) | 95,617 (1,471) | 92,038 (804) | 82,751 (5,710) | 89,526 (3,672) |
| Clinical Specialty - Primary Care^[*]^ | 21.1 (0.5) | 3.4 (0.3) | 37.5 (1) | 19.2 (2.7) | 21.4 (2.4) |
| Use telehealth at work | 27 (1.0) | 36.1 (1.9) | 17.9 (0.7) | 19.9 (2.5) | 34.1 (3.8) |
| Satisfied with job | 89 (1.0) | 88.4 (1.9) | 89.9 (0.5) | 88.5 (2.2) | 88.1 (4.1) |
| Extremely satisfied with job | 44.7 (1.0) | 44.2 (1.7) | 45.4 (1.0) | 49.3 (5) | 40.5 (3.3) |
| Have considered leaving current job? | 54.8 (1.0) | 58.6 (1.8) | 52.5 (1.1) | 53.5 (2.9) | 51 (4.5) |
| Female | 90.7 (0.6) | 90.4 (0.9) | 92 (0.5) | 86.7 (7.1) | 89.7 (1.4) |
| Race: Non-Hispanic White | 72.1 (1.0) | 70.9 (2.0) | 74.4 (0.9) | 70 (6.1) | 68.8 (5.1) |
| Hours Worked | 1,971 (18) | 2,023 (20) | 1,942 (13) | 1,953 (88) | 1,910 (86) |
| Full Time Worker (1,820+ hours) | 70.5 (1.2) | 76.8 (1.5) | 66 (1.1) | 66 (5.7) | 68.6 (5.6) |
| NP Experience (years) | 8.2 (0.2) | 7.2 (0.2) | 8.7 (0.2) | 8.8 (0.8) | 9.5 (0.6) |
| NP Education: Master’s | 81.9 (1) | 82.6 (1.8) | 84.2 (0.7) | 71.8 (6) | 77.5 (2.7) |
| NP Education: Doctorate | 5.1 (0.6) | 4.3 (0.6) | 4.4 (0.3) | 11.9 (7.2) | 6.1 (1.4) |
| Care Area-Specific National Certification: Family | 53.5 (0.6) | 40.8 (1.1) | 64.9 (0.7) | 42.7 (1.9) | 49.8 (1.7) |
| Care Area-Specific National Certification: None | 4.3 (0.4) | 5.3 (0.9) | 1.9 (0.2) | 12.3 (2.0) | 6.4 (1.1) |
| Feel that you are NOT able to practice to full SOP | 11.3 (0.4) | 13.9 (0.8) | 8.5 (0.4) | 15.2 (1.5) | 15.6 (1.5) |
| Feel that you are NOT able to practice to full NP education | 10.4 (0.4) | 11.7 (0.7) | 8.7 (0.5) | 12.6 (1.7) | 14.2 (1.4) |
| Have a patient panel | 47.9 (0.6) | 34.6 (1.0) | 54.9 (0.7) | 58.2 (2.6) | 49.2 (1.9) |
| Size of panel (# of patients) | 449 (10) | 260 (18) | 559 (14) | 187 (19) | 400 (28) |
| Job Covered by Union | 11.6 (0.9) | 16.1 (1.9) | 6 (0.4) | 3.3 (0.8) | 22.3 (4) |
| First Nursing Education - Associate's degree | 30.6 (1) | 30.1 (2) | 28 (0.9) | 37 (3.8) | 38.4 (4.1) |
| No prior health-related job | 31.3 (1.0) | 30.6 (1.6) | 33 (1.0) | 28.3 (3.5) | 29.2 (4.5) |
| Married | 75.7 (1.0) | 71.4 (1.8) | 80.1 (0.8) | 74 (3.1) | 75.3 (3.1) |
| Has Young (<6) Children | 23.9 (1.0) | 23.9 (1.3) | 24.5 (0.7) | 26.1 (6.4) | 20.4 (5) |
| Has Older (6-18) Children | 36 (0.8) | 33.5 (1.5) | 37.6 (1.1) | 39.2 (5.3) | 36.7 (4.6) |
| % of time spent during work - Management, Supervision, and Admin | 9.8 (0.5) | 10.6 (0.9) | 6.4 (0.3) | 12.6 (1.6) | 17.2 (3.2) |
| % of time spent during work - non-nursing tasks (eg, housekeeping, locating supplies) | 2.4 (0.1) | 3.0 (0.2) | 1.8 (0.1) | 2.2 (0.3) | 2.8 (0.9) |
| % of time spent during work - Patient care | 66.6 (0.6) | 63.6 (1.1) | 76.2 (0.6) | 63.1 (2.1) | 42.7 (2.4) |
| Average number of patients seen per week | 57.1 (0.4) | 48.8 (0.7) | 65.4 (0.5) | 45.2 (1.5) | 44.3 (1.8) |
| Worked for current employer >=5 years | 46.8 (0.8) | 57.1 (1.8) | 40.1 (1.0) | 34.6 (3.7) | 43.4 (3.8) |
| Employment main task (>50% of time) is patient care / charting | 73.1 (1.1) | 68.5 (1.8) | 86.3 (0.9) | 69.7 (3.5) | 41.9 (3.2) |
| Desired more training - Mental health | 32.4 (0.9) | 26.7 (2) | 38.8 (0.9) | 30.4 (3.4) | 29.8 (2.9) |
| Desired more training - Value-based care | 16.4 (0.9) | 19.8 (2) | 14.5 (0.5) | 15.1 (2.2) | 12.4 (1.5) |
| Ever Licensed as LPN/LVN | 11.2 (0.5) | 10.4 (1.0) | 10 (0.5) | 19.8 (2.8) | 13.5 (1.7) |
| Geo: East North Central | 16 (0.8) | 17.9 (1.5) | 14 (0.7) | 16.3 (2.2) | 16.5 (4.9) |
| Geo: East South Central | 8.5 (0.3) | 8 (0.8) | 9.9 (0.5) | 6.1 (1.1) | 6.8 (0.7) |
| Geo: Middle Atlantic | 13 (0.5) | 15.4 (1.0) | 10.4 (0.5) | 16.2 (2.1) | 12 (1.6) |
| Geo: Mountain | 6.7 (0.3) | 5.8 (0.6) | 7.4 (0.2) | 7 (1.1) | 7 (0.8) |
| Geo: New England | 6.3 (0.4) | 6.1 (0.7) | 6.6 (0.4) | 5.5 (0.8) | 6.3 (1.3) |
| Geo: Pacific | 12.5 (0.9) | 12.6 (1.8) | 12 (0.6) | 7.3 (1.2) | 17.6 (4.8) |
| Geo: South Atlantic | 20.6 (0.7) | 18.7 (1.1) | 21.3 (0.7) | 27.4 (6.1) | 20.1 (2.3) |
| Geo: West North Central | 7.6 (0.4) | 8.1 (0.8) | 7.6 (0.5) | 6.7 (1.4) | 6.4 (1.3) |
| Geo: West South Central | 8.9 (0.3) | 7.5 (0.6) | 10.9 (0.5) | 7.5 (1.6) | 7.3 (0.9) |
| Age: under 35 | 18.8 (0.7) | 21.5 (1.5) | 19.8 (0.8) | 9.5 (1.6) | 11.3 (2.6) |
| Age: 35-44 | 31.4 (0.9) | 33 (1.9) | 31.4 (1.0) | 25.2 (2.9) | 29.7 (5.1) |
| Age: 45-54 | 25.3 (0.9) | 25.2 (1.8) | 24.7 (0.7) | 29.9 (5.9) | 24.6 (2.6) |
| Age: 55-64 | 18.7 (0.6) | 16.8 (1.0) | 18 (0.5) | 26.2 (3.0) | 23.8 (2.5) |
| Age: 65 over | 5.9 (0.2) | 3.5 (0.3) | 6.2 (0.4) | 9.1 (2.0) | 10.7 (1.2) |
| **SOURCE** Authors’ analysis of 2018 NSSRN data. **NOTES** Sample means followed by standard errors (adjusted for complex survey design).  * = There is no uniform definition of primary care in different data sets, so there may be variation in results compared to other surveys. | | | | | |

| **Appendix Table A3: Full Main Regression Results** | | | |
| --- | --- | --- | --- |
|  | Basic Model | Intermediate Model | Full Model |
| (Intercept) | 95,616.586*** | -5,766.887+ | -5,278.875 |
|  | (1,471.231) | (2,925.854) | (4,501.164) |
| work_set_groupClinic / Ambulatory | -3,578.791* | -635.826 | -950.342 |
|  | (1,551.126) | (1,202.741) | (1,120.976) |
| work_set_groupLong-term Care | -12,865.373* | -6,984.809* | -6,777.290* |
|  | (5,786.940) | (2,992.281) | (2,897.787) |
| work_set_groupOther Setting | -6,090.263+ | -1,840.803 | -2,139.595 |
|  | (3,481.079) | (1,707.978) | (1,712.868) |
| primary_hours |  | 68.696*** | 60.240*** |
|  |  | (2.319) | (2.601) |
| I(primary_hours^2) |  | -0.010*** | -0.009*** |
|  |  | (0.001) | (0.001) |
| census_divisionEast North Central |  | -2,572.663 | -2,473.948 |
|  |  | (1,976.408) | (1,862.242) |
| census_divisionMountain |  | 4,897.004* | 3,952.780* |
|  |  | (2,009.346) | (1,716.857) |
| census_divisionPacific |  | 23,033.629*** | 21,487.370*** |
|  |  | (2,649.835) | (2,626.116) |
| census_divisionSouth Atlantic |  | 1,400.948 | 588.294 |
|  |  | (2,110.960) | (1,968.288) |
| census_divisionNew England |  | 6,298.696** | 5,885.192** |
|  |  | (2,080.979) | (1,830.988) |
| census_divisionMiddle Atlantic |  | 9,861.355*** | 8,380.852*** |
|  |  | (1,703.857) | (1,522.919) |
| census_divisionWest South Central |  | 9,217.299*** | 8,452.528*** |
|  |  | (1,942.455) | (1,764.687) |
| census_divisionEast South Central |  | -4,213.415* | -4,840.477** |
|  |  | (1,972.997) | (1,813.132) |
| female |  |  | -5,566.502* |
|  |  |  | (2,582.015) |
| full_time_work |  |  | 8,181.916*** |
|  |  |  | (1,775.434) |
| experience_yrs_np |  |  | 1,894.640*** |
|  |  |  | (213.341) |
| I(experience_yrs_np^2) |  |  | -51.659*** |
|  |  |  | (6.283) |
| np_educationBACHELOR'S OR CERTIFICATE/AWARD |  |  | -3,183.357 |
|  |  |  | (3,217.730) |
| np_educationPOST MASTER'S CERTIFICATE |  |  | 1,637.368 |
|  |  |  | (1,989.201) |
| np_educationDOCTORATE |  |  | 1,322.830 |
|  |  |  | (4,453.219) |
| time_spent_patcare |  |  | 15.783 |
|  |  |  | (25.676) |
| married |  |  | 1,128.674 |
|  |  |  | (1,083.396) |
| union_job |  |  | 1,040.795 |
|  |  |  | (1,817.549) |
| children_young |  |  | -1,001.581 |
|  |  |  | (1,993.887) |
| children_not_young |  |  | -1,469.506 |
|  |  |  | (1,224.470) |
| race_nh_white |  |  | -235.262 |
|  |  |  | (1,465.571) |
| LPNVN_license |  |  | -897.240 |
|  |  |  | (1,807.437) |
| prev_job__no_health |  |  | 293.549 |
|  |  |  | (1,150.760) |
| current_job_5yrs |  |  | 617.616 |
|  |  |  | (991.185) |
| age_bins35-44 |  |  | 4,967.909** |
|  |  |  | (1,528.298) |
| age_bins45-54 |  |  | 3,596.852* |
|  |  |  | (1,778.088) |
| age_bins55-64 |  |  | 1,916.658 |
|  |  |  | (1,808.615) |
| age_bins65 over |  |  | -2,800.896 |
|  |  |  | (2,195.733) |
| N | 21,266 | 21,266 | 21,266 |
| R^2 | 0.01 | 0.43 | 0.46 |
| F-statistic | 3.2 | 183.3 | 106.3 |
| + p < 0.1, * p < 0.05, ** p < 0.01, *** p < 0.001 | | | |

There were some errors when calculating the F-statistic in R, so these values are from the same regression but run with Stata version 17, which had the same estimates and coefficients as the models in R.

| **Appendix Table A4: Robustness Regression Results, Part 1** | | | | | | |
| --- | --- | --- | --- | --- | --- | --- |
|  | Total: Log | Total: + Scope of Practice | Hourly: Raw | Hourly: Winzorized (0.1%) | Hourly: Log | Hourly: Log, removing hours worked |
| (Intercept) | 9.144*** | -8,591.118 | 124.994*** | 124.994*** | 4.006*** | 3.696*** |
|  | (0.112) | (5,556.269) | (16.764) | (16.764) | (0.129) | (0.073) |
| work_set_groupClinic / Ambulatory | -0.013 | -888.314 | 1.776 | 1.776 | 0.011 | 0.000 |
|  | (0.015) | (1,112.276) | (1.412) | (1.412) | (0.017) | (0.016) |
| work_set_groupLong-term Care | -0.133* | -6,731.249* | -3.864 | -3.864 | -0.112 | -0.123+ |
|  | (0.066) | (2,901.589) | (3.502) | (3.502) | (0.075) | (0.073) |
| work_set_groupOther Setting | -0.023 | -2,129.060 | -1.031 | -1.031 | -0.010 | -0.001 |
|  | (0.032) | (1,727.897) | (3.061) | (3.061) | (0.025) | (0.029) |
| female | -0.013 | -5,545.687* | -1.509 | -1.509 | -0.009 | -0.002 |
|  | (0.050) | (2,596.439) | (2.476) | (2.476) | (0.056) | (0.054) |
| primary_hours | 0.002*** | 60.275*** | -0.074*** | -0.074*** | -0.000** |  |
|  | (0.000) | (2.603) | (0.014) | (0.014) | (0.000) |  |
| I(primary_hours^2) | -0.000*** | -0.009*** | 0.000*** | 0.000*** | -0.000 |  |
|  | (0.000) | (0.001) | (0.000) | (0.000) | (0.000) |  |
| full_time_work | -0.070* | 8,179.067*** | 20.293*** | 20.293*** | 0.158*** | -0.096** |
|  | (0.035) | (1,773.131) | (3.758) | (3.758) | (0.032) | (0.028) |
| experience_yrs_np | 0.022*** | 1,898.419*** | 1.026* | 1.026* | 0.022*** | 0.018*** |
|  | (0.004) | (212.528) | (0.415) | (0.415) | (0.004) | (0.004) |
| I(experience_yrs_np^2) | -0.001*** | -51.832*** | -0.023 | -0.023 | -0.001*** | -0.000*** |
|  | (0.000) | (6.265) | (0.017) | (0.017) | (0.000) | (0.000) |
| np_educationBACHELOR'S OR CERTIFICATE/AWARD | -0.030 | -3,201.186 | -7.290* | -7.290* | -0.067 | -0.061 |
|  | (0.048) | (3,218.199) | (3.356) | (3.356) | (0.041) | (0.043) |
| np_educationPOST MASTER'S CERTIFICATE | 0.030 | 1,659.919 | 0.782 | 0.782 | 0.023 | 0.024 |
|  | (0.026) | (2,003.588) | (2.018) | (2.018) | (0.028) | (0.029) |
| np_educationDOCTORATE | -0.055 | 1,322.552 | -4.544 | -4.544 | -0.069 | -0.070 |
|  | (0.100) | (4,467.747) | (5.049) | (5.049) | (0.112) | (0.105) |
| time_spent_patcare | 0.001* | 15.124 | -0.028 | -0.028 | 0.000 | 0.001 |
|  | (0.000) | (25.379) | (0.042) | (0.042) | (0.000) | (0.000) |
| married | 0.004 | 1,140.636 | 0.763 | 0.763 | 0.011 | 0.009 |
|  | (0.017) | (1,083.981) | (1.414) | (1.414) | (0.018) | (0.019) |
| union_job | 0.033 | 941.360 | -0.483 | -0.483 | 0.022 | 0.035 |
|  | (0.022) | (1,796.500) | (1.962) | (1.962) | (0.022) | (0.021) |
| children_young | -0.029 | -1,021.432 | -2.815 | -2.815 | -0.037 | -0.022 |
|  | (0.038) | (1,993.330) | (2.102) | (2.102) | (0.041) | (0.040) |
| children_not_young | -0.013 | -1,483.256 | -1.181 | -1.181 | -0.019 | -0.010 |
|  | (0.021) | (1,223.719) | (1.335) | (1.335) | (0.022) | (0.023) |
| race_nh_white | -0.003 | -116.580 | -1.330 | -1.330 | 0.001 | 0.000 |
|  | (0.023) | (1,461.803) | (2.125) | (2.125) | (0.025) | (0.025) |
| LPNVN_license | 0.011 | -867.420 | 0.139 | 0.139 | 0.005 | 0.008 |
|  | (0.028) | (1,804.769) | (1.526) | (1.526) | (0.029) | (0.029) |
| prev_job__no_health | 0.001 | 304.920 | -0.977 | -0.977 | -0.001 | 0.008 |
|  | (0.019) | (1,150.894) | (1.540) | (1.540) | (0.020) | (0.020) |
| current_job_5yrs | -0.003 | 616.677 | -0.236 | -0.236 | 0.007 | 0.004 |
|  | (0.014) | (985.248) | (1.175) | (1.175) | (0.015) | (0.015) |
| age_bins35-44 | 0.069** | 4,990.775** | 3.393* | 3.393* | 0.069** | 0.060** |
|  | (0.023) | (1,529.123) | (1.359) | (1.359) | (0.022) | (0.023) |
| age_bins45-54 | 0.034 | 3,623.949* | 1.149 | 1.149 | 0.025 | 0.014 |
|  | (0.035) | (1,769.523) | (1.996) | (1.996) | (0.039) | (0.037) |
| age_bins55-64 | 0.034 | 1,934.958 | 0.618 | 0.618 | 0.021 | 0.014 |
|  | (0.033) | (1,807.395) | (2.069) | (2.069) | (0.034) | (0.032) |
| age_bins65 over | -0.104* | -2,689.571 | -0.308 | -0.308 | -0.049 | -0.022 |
|  | (0.044) | (2,194.225) | (4.141) | (4.141) | (0.047) | (0.050) |
| census_divisionEast North Central | -0.015 | -2,786.204 | -0.451 | -0.451 | -0.018 | -0.024 |
|  | (0.035) | (1,892.988) | (1.853) | (1.853) | (0.036) | (0.036) |
| census_divisionMountain | 0.035 | 5,449.172* | 2.475 | 2.475 | 0.051+ | 0.049+ |
|  | (0.031) | (2,423.566) | (2.233) | (2.233) | (0.030) | (0.027) |
| census_divisionPacific | 0.227*** | 21,685.089*** | 13.498*** | 13.498*** | 0.229*** | 0.242*** |
|  | (0.034) | (2,736.155) | (3.024) | (3.024) | (0.037) | (0.033) |
| census_divisionSouth Atlantic | 0.006 | 456.175 | 1.562 | 1.562 | 0.010 | 0.011 |
|  | (0.035) | (1,978.141) | (2.347) | (2.347) | (0.037) | (0.035) |
| census_divisionNew England | 0.077* | 5,776.775** | 4.339* | 4.339* | 0.083** | 0.064* |
|  | (0.029) | (1,839.321) | (2.110) | (2.110) | (0.029) | (0.028) |
| census_divisionMiddle Atlantic | 0.119*** | 8,077.066*** | 9.007** | 9.007** | 0.128*** | 0.131*** |
|  | (0.024) | (1,760.178) | (2.634) | (2.634) | (0.024) | (0.023) |
| census_divisionWest South Central | 0.121*** | 8,137.516*** | 5.602** | 5.602** | 0.114*** | 0.115*** |
|  | (0.026) | (1,984.667) | (1.997) | (1.997) | (0.027) | (0.026) |
| census_divisionEast South Central | -0.048 | -5,170.551* | -0.819 | -0.819 | -0.053+ | -0.052+ |
|  | (0.029) | (1,951.688) | (2.089) | (2.089) | (0.030) | (0.030) |
| sop_levelPartial |  | 3,592.401 |  |  |  |  |
|  |  | (2,512.768) |  |  |  |  |
| sop_levelRestricted |  | 3,454.267 |  |  |  |  |
|  |  | (3,336.875) |  |  |  |  |
| N | 21,266 | 21,266 | 21,266 | 21,266 | 21,266 | 21,266 |
| R^2 | 0.60 | 0.47 | 0.12 | 0.12 | 0.17 | 0.10 |
| + p < 0.1, * p < 0.05, ** p < 0.01, *** p < 0.001 | | | | | | |

| **Appendix Table A5: Robustness Regression Results, Part 2** | | |
| --- | --- | --- |
|  | Full Sample (main model) | Removing Hospital-Based Ambulatory Care |
| (Intercept) | -5,278.875 | -5,015.256 |
|  | (4,501.164) | (4,755.584) |
| work_set_groupClinic / Ambulatory | -950.342 | -1,323.227 |
|  | (1,120.976) | (1,372.800) |
| work_set_groupLong-term Care | -6,777.290* | -7,265.668* |
|  | (2,897.787) | (2,970.106) |
| work_set_groupOther Setting | -2,139.595 | -2,458.706 |
|  | (1,712.868) | (1,929.257) |
| female | -5,566.502* | -5,182.412+ |
|  | (2,582.015) | (2,698.185) |
| primary_hours | 60.240*** | 60.355*** |
|  | (2.601) | (2.725) |
| I(primary_hours^2) | -0.009*** | -0.009*** |
|  | (0.001) | (0.001) |
| full_time_work | 8,181.916*** | 7,594.372*** |
|  | (1,775.434) | (1,904.973) |
| experience_yrs_np | 1,894.640*** | 1,877.111*** |
|  | (213.341) | (228.340) |
| I(experience_yrs_np^2) | -51.659*** | -52.671*** |
|  | (6.283) | (6.722) |
| np_educationBACHELOR'S OR CERTIFICATE/AWARD | -3,183.357 | -2,286.734 |
|  | (3,217.730) | (3,432.351) |
| np_educationPOST MASTER'S CERTIFICATE | 1,637.368 | 2,004.416 |
|  | (1,989.201) | (2,123.007) |
| np_educationDOCTORATE | 1,322.830 | 945.433 |
|  | (4,453.219) | (4,709.287) |
| time_spent_patcare | 15.783 | 11.382 |
|  | (25.676) | (28.463) |
| married | 1,128.674 | 1,314.730 |
|  | (1,083.396) | (1,112.305) |
| union_job | 1,040.795 | 104.300 |
|  | (1,817.549) | (2,020.061) |
| children_young | -1,001.581 | -1,058.247 |
|  | (1,993.887) | (2,146.808) |
| children_not_young | -1,469.506 | -1,362.673 |
|  | (1,224.470) | (1,292.791) |
| race_nh_white | -235.262 | -1.604 |
|  | (1,465.571) | (1,606.933) |
| LPNVN_license | -897.240 | -843.963 |
|  | (1,807.437) | (1,925.244) |
| prev_job__no_health | 293.549 | 146.722 |
|  | (1,150.760) | (1,216.308) |
| current_job_5yrs | 617.616 | 1,037.609 |
|  | (991.185) | (1,078.325) |
| age_bins35-44 | 4,967.909** | 5,623.145** |
|  | (1,528.298) | (1,637.126) |
| age_bins45-54 | 3,596.852* | 4,224.348* |
|  | (1,778.088) | (1,937.413) |
| age_bins55-64 | 1,916.658 | 2,037.083 |
|  | (1,808.615) | (1,958.553) |
| age_bins65 over | -2,800.896 | -2,086.480 |
|  | (2,195.733) | (2,294.301) |
| census_divisionEast North Central | -2,473.948 | -2,977.001 |
|  | (1,862.242) | (1,843.412) |
| census_divisionMountain | 3,952.780* | 3,146.209+ |
|  | (1,716.857) | (1,683.578) |
| census_divisionPacific | 21,487.370*** | 20,497.981*** |
|  | (2,626.116) | (2,688.448) |
| census_divisionSouth Atlantic | 588.294 | 127.366 |
|  | (1,968.288) | (2,038.235) |
| census_divisionNew England | 5,885.192** | 5,386.522** |
|  | (1,830.988) | (1,952.264) |
| census_divisionMiddle Atlantic | 8,380.852*** | 8,232.522*** |
|  | (1,522.919) | (1,507.569) |
| census_divisionWest South Central | 8,452.528*** | 7,783.661*** |
|  | (1,764.687) | (1,687.712) |
| census_divisionEast South Central | -4,840.477** | -5,543.629** |
|  | (1,813.132) | (1,792.468) |
| N | 21,266 | 19,165 |
| R^2 | 0.46 | 0.46 |
